# Navigating the Pathway to Care in Adults With Cerebral Palsy

**DOI:** 10.3389/fneur.2021.734139

**Published:** 2021-09-16

**Authors:** Edward A. Hurvitz, Daniel G. Whitney, Brigid Waldron-Perrine, Dayna Ryan, Heidi J. Haapala, Mary Schmidt, Cathryn Gray, Mark D. Peterson

**Affiliations:** ^1^Department of Physical Medicine and Rehabilitation, Michigan Medicine, University of Michigan, Ann Arbor, MI, United States; ^2^Institute for Healthcare Policy and Innovation, Michigan Medicine, University of Michigan, Ann Arbor, MI, United States

**Keywords:** cerebral palsy, health systems, care coordination, telemedicine, mental health

## Abstract

As individuals with cerebral palsy (CP) age, they face unique challenges which complicate their ability to access and receive appropriate health care. These problems exist at the level of the health care system, the clinician, and the individual. At the system level, there is an inadequate number of professionals who are informed of and interested in the care of adults with CP. Pediatric clinicians prefer treating children, and adult caregivers are not knowledgeable about and may feel less competent about CP. Pediatric care does not translate well to the adult population, and information about best practices for adults is just starting to develop. Differences in the physiologic development of individuals with CP render well-established clinical protocols for risk screening of chronic diseases less effective. Moreover, lack of supportive resources decreases a caregiver's sense of self-efficacy in treating this population. The patient's ability to navigate these barriers is complicated by the high prevalence of comorbid cognitive impairment and mental health issues including anxiety, depression, and other psychiatric disorders; a bidirectional relationship between challenges in navigating care/needs and comorbid mental health conditions appears likely. Many patients have additional barriers related to social determinants of health, such as access to transportation, accessible health care facilities, and other personal and environmental factors that may impede health maintenance and wellness. Increasing and disseminating knowledge, harnessing the power of new technologies such as telemedicine, and addressing mental health issues are some of the methods that are available to help adults with CP navigate this road.

## Introduction

Cerebral palsy (CP) arises early in life and is historically viewed as a non-progressive pediatric condition affecting movement, muscle tone, or posture. With improvements in care, individuals with CP are living well into adulthood (1). It follows, then, that there are more adults than children with CP, indicating that CP is also an adult condition. In health care systems, however, CP is often confined to pediatric hospitals and ambulatory care services, while adults with CP struggle to receive appropriate care, even for the most common disorders such as musculoskeletal morbidities (2). As individuals with CP age, they face unique challenges which make their medical care complex. Models of care and interventions that worked for them as children do not exist and may not be appropriate for them as adults, owing in part to new problems that arise with aging. Consequently, medical care for adults with CP requires different expertise and coordinated approaches. The complex health care pathway that adults with CP must navigate poses challenges at the level of the health care system, the clinical provider, and within the individuals themselves.

### Health Care Systems

There are an inadequate number and network of health care providers who have both the interest and the expertise to provide appropriate care for adults with CP. Adults with CP have complex issues requiring longer appointments which are hard to fit into busy clinical schedules and make care for this population less appealing for a health system. Additionally, there is a significant knowledge gap about the unique issues facing this population. Most professional schools provide little training in the care of individuals with disabilities. People who grow up with pediatric-onset disabilities form an even smaller “niche” population, and even less exposure within curriculums. Compounding this problem is the general lack of published scientific guidance in this area. The literature on adults with CP has grown significantly in the past several years (1), and has focused on identifying and defining problems across the lifespan; however, few studies have informed the implementation for proper screening and intervention protocols.

Adults with CP often find themselves in pediatric models of care. Many receive care in pediatric hospitals (3) or in pediatric clinical settings that have, at best, an “adult day.” Finding the right specialists to staff these clinics is a conundrum: many pediatric providers do not want to deal with the complex medical and psychosocial issues of adults, while adult providers are not comfortable with a diagnosis that they were exposed to briefly during their medical school pediatric rotation. The pediatric clinic model with the typical multidisciplinary milieu (physiatry, orthopedics, neurosurgery, therapies) does not address some of the most pressing issues of the adult CP population, which may include multimorbidity (4, 5) across organ systems spanning cardiac, renal, pulmonary, musculoskeletal and mental health issues (6). In general adult care, multidisciplinary clinics are less common because there are so many varied needs, and because busy, independent adults don't have the time for the typical multidisciplinary 4 hour appointment.

### Clinical Providers

Providers with these knowledge gaps will have poor self-efficacy related to the care of adults with CP. Even physiatrists, who are trained to care for individuals with disabilities, struggle with trying to apply information from their pediatric and stroke rotations to a population who represents neither of those. For surgical providers, care provision is more complicated due to a lack of experience and resultant lack of knowledge of potential risks, outcomes, and best post-operative protocols. Knowledge development is greatly needed in several areas related to preventive care and the treatment of medical and rehabilitation needs of adults with CP.

Preventive care is a high priority for adults with CP, but there are several differences from the general population which are most likely not known by most providers. Adults with CP are at risk for multimorbidity at a much younger age than their non-CP peers. Among young adults with CP (i.e., 18–30 years), 1 in 4 has multimorbidity (5). Despite this, adults with CP are less likely to receive preventive care (7). A large, nationwide study examining the natural history of several morbidities across the adult lifespan recommended: “Broadly speaking, whenever feasible, preventive and monitoring measures should be adopted early in the lifespan and not later than age 50 years for adults with cerebral palsy” (8). To achieve a successful systematic approach for earlier screening of comorbidity risk, considerable efforts will be needed to reform primary prevention for adults with CP with investments from a variety of stakeholders (e.g., clinical systems, insurance providers, clinicians, patients, policy makers, etc.).

As a result of living with a lifelong physical disability, individuals with CP have alterations in muscle and bone development. The smaller and unique body sizes and heterogeneity in muscle deficits across the body (9) can result in erroneous interpretation of commonly used approaches [e.g., body mass index (BMI)] when assessing body habitus and obesity risk (10, 11). Clinical interpretation of obesity risk from body fat, whether total body or regional fat depots, can be misleading and inconsistent based on the approach used and anatomical site examined in persons with CP [e.g., normal BMI but high visceral fat found with imaging (12)]. BMI can underestimate total body fat, with the degree of underestimation being greater for more severe forms of CP (13). This is largely due to the lower fat-free mass in CP, which reduces the numerator in the BMI ratio-thus lowering the BMI value independent of fat mass. Additionally, fat may accumulate disproportionately among visceral and musculoskeletal depots (14, 15), despite “normal” subcutaneous fat stores and even normal BMI-calling into question the utility of subcutaneous-derived methods to assess body fat, such as skinfold measurements. Therefore, clinicians may miss opportunities for detection of obesity risk and its associated disease sequela. Adults with CP have significantly higher rates of risk factors for metabolic syndrome with resultant risk of cardiovascular disease, stroke, and other chronic diseases related to excess body fat (16–18), and low levels of fitness and physical activity (19), so appropriate screening is critical.

Another example is screening for chronic kidney disease, which is prevalent in adults with CP (16, 20–23). Kidney disease is assessed by glomerular filtration rate (GFR), which is a measure of kidney function. Measuring GFR is the gold-standard for GFR assessment, but is cumbersome, so GFR is often estimated using serum biomarkers (eGFR), including creatinine and/or cystatin c (24–26). The problem for interpreting eGFR in CP is that creatinine and cystatin c are influenced by the amount of muscle and fat mass, respectively, which can lead to inaccurate interpretation of kidney function and risk of kidney disease (27–29). Creatinine-based eGFR is more commonly performed clinically and is typically part of routine blood work. Individuals with CP have lower levels of creatinine due to reduced muscle, which artificially inflates eGFR values and leads to a false interpretation of healthier kidney function.

Bone fragility and fractures are common in CP due to a variety of interacting factors (30–34), and are associated with significant morbidity and early mortality (35). Bone strength is often monitored clinically by the sole interpretation of bone mineral density (BMD) from dual-energy x-ray absorptiometry. However, BMD alone may not be sufficient to assess bone strength or bone fragility for adults with CP. BMD is a ratio of bone mineral content relative to bone area. The smaller bones in CP (36, 37) can inflate BMD independent of bone mineral content, presenting clinically as a bone that is stronger than it actually is. Further, bone size is itself an independent determinant of fracture risk (38, 39), which is missed when assessing BMD alone.

In addition to these complexities of health screening, the adult with CP brings many unique challenges into the clinic to providers unfamiliar with this population. A major one is early functional loss. Mobility decreases early in adulthood for many individuals with CP, sometimes in the fourth decade for those with bilateral involvement (40). This functional loss is often attributed to pain and fatigue. Many adults with CP are told that their “CP is getting worse.” Clinicians (and patients) must understand that functional loss is not a “progression of CP,” nor just a result of pain and fatigue, but rather a product of several factors, many of which are treatable, including poor fitness levels, new neurologic problems, and the effects of aging and chronic disease. As an example, there is a higher incidence of myelopathy and stroke in CP which may go undiagnosed and contribute to functional loss (41).

Pain is common in adults with CP. Recent studies showed that up to 70% of adults with CP experience pain, which can affect life activities and sleep, and increase risk for psychiatric disorders (42–44). However, pain cannot be addressed and treated as it is in the general population. First, it is most likely under-reported in individuals with communication difficulties, making it complex to assess (42). In addition, most practitioners would assume that adults with CP have nociceptive (i.e., peripheral) pain based on their history of poor biomechanics, joint dysplasias, muscle spasticity, and prior surgeries. They may easily miss the fact that many adults with CP have a heterogeneous pain phenotype, which could also include nociplastic pain or a mixed picture (45, 46). Prescription of opioids for pain is significantly higher in this population, an indication that their healthcare providers struggle with finding appropriate treatment options (45). Thus, increasing clinical awareness of the pain phenotypes, improving clinical pain screening strategies, and developing efficient referral resources for appropriate pain management may help reduce the burden of opioid addiction and overdose in these population.

Physical and occupational therapists working with adults with CP must adapt as well. Adults with CP is that they have never moved “normally” in their lifetime, which creates a challenge for their therapists who typically focus on helping patient's return to “normal” or near-normal function. Throughout life, persons with CP have had to overcome some amount of weakness and/or hypertonicity whenever they move, which has resulted in movement patterns that are not biomechanically appropriate or efficient. Adults with CP may not know how to volitionally contract a single muscle or isolate a muscle contraction without utilizing a spasticity pattern in the entire limb. Additionally, abnormal joint reaction forces as a consequence of muscle spasticity and atypical movement patterns accelerate the “aging” process of the joints, resulting in increased incidence of orthopedic conditions such as osteoarthritis (47).

Finding an appropriate rehabilitation plan for this complex patient population can be challenging. Pain, spasticity, and multiple musculoskeletal problems interfere with therapy. Many of these patients require orthotics and/or use wheelchairs, so deep knowledge of these assistive devices is necessary when treating this population. Unfortunately, insurance coverage limitations often interfere with maximizing rehabilitation interventions. Many adults with CP may only get 6–12 visits for physical and occupational therapy for an entire year, regardless of their functional limitations and potential to benefit from rehabilitation intervention. Insurance companies commonly only cover a new wheelchair or orthotic every 5 years; thus, it is very important that the correct, most useful device be ordered initially. Given the importance of accurate and timely care, it is vital for primary care physicians to collaborate with physical therapists or physiatrists who specialize in assistive devices prior to ordering equipment to ensure that the patient's needs are met.

### The Individual

Several individual factors related to the diagnosis of CP in adults can function as barriers to optimizing physical health and accessing appropriate care. Many secondary health complications occur in adults with CP due to immobility. Despite their physical impairments, many patients may try to exercise and improve their physical activity but then encounter equipment that is logistically or financially inaccessible, resulting in decreased engagement in proactive positive health behaviors such as exercise. Similarly, logistical barriers exist (e.g., transportation, flexible scheduling) to accessing optimal physical health care. Even for patients who want to do the right thing, there are many obstacles to negotiate.

Mental health challenges can also function as barriers to optimal health care and, subsequently, health related quality of life (although, it is important to note that the associations between mental health challenges with medical co-morbidities, function, societal and psychosocial factors can be bidirectional and complex) (48). Adults with CP have a greater prevalence of mental health disorders including mood and anxiety disorders as well as thought disorder, personality or behavioral disorders, and substance use disorders, as compared to the general population (49, 50). In the context of reported declines in mobility status in persons with CP as they reach adulthood, mobility decline has been linked to worse mental health status, with potential mechanisms including functional loss, pain, fatigue, and reduced participation (51).

Although most research has focused on the influence of physical function on mental health, it is also important to consider the potential influence of mental health functioning on self-care and self-management of chronic physical challenges. The broad mental health literature clearly demonstrates that mental illness can negatively impact compliance with the plan of care and recommended interventions (52, 53). Negative influence of mental health disorders on individual's engagement in self-management is well-acknowledged in the rehabilitation literature (54, 55). Over time, mental health factors may cause or exacerbate a learned helplessness associated with chronic difficulties and may be associated with lower levels of resilience. Addressing mental health needs may result in health re-prioritization and subsequent deference of some aspects of physical care as emotional needs become primary.

An additional barrier to optimal care of persons with CP is the well-established fact that persons with various types of disabilities are at a disadvantage broadly within our healthcare system. This disadvantage is related to both understanding of health needs and socioeconomic and community-based factors that influence access to and utility of healthcare approaches (56, 57). Equitable access for persons with CP to meet their needs can include accessible transportation, housing, and health care facilities, and employment opportunities that are flexible enough to accommodate the varied practical and medical needs of persons with disabilities. Adults with CP may lack the resources needed to acquire greater resources, resulting in a cycle of inadequate support that perpetuates ongoing health and functional declines.

### Navigating the Path Forward

The first step in addressing the many roadblocks on the way to appropriate health care for adults with CP is knowledge development, transfer, and translation. Individuals with CP need greater knowledge about the complexities and unique aspects of their care to increase their self-efficacy in their self-management. Healthcare providers in multiple medical, therapy, and other disciplines need access to training in evidence-based care, and that evidence must continue to be developed. Long-term studies and health surveillance of CP using registries are critical for this goal (58). On a system-wide level, there is a need for greater knowledge translation aimed at advocacy services such as coordinated care options, patient navigators, and value-based care models that would make care for adults with CP efficient and more attractive to providers (1).

New technologies bring options for models of care that differ from pediatric clinics and address the unique needs of this population. Issues related to health promotion can be addressed through telemedicine. Knowledgeable clinicians can connect with patients who live at a distance from their center and work virtually with their local providers on issues that need to be addressed in person, contributing expertise and guidance. The medical home for an adult with CP needs a provider network that incorporates many specialists beyond those classically attending the “CP clinic,” including cardiologists, endocrinologists, nephrologists, psychiatrists, and many others trained in adult CP care. Telemedicine can bring together patients and specialists, creating virtual medical homes. Clinicians reported high satisfaction with telemedicine in a study looking at its use in children with CP (59). It has been used successfully in many neurologic conditions for adults (60) and to provide care for several problems common in adults with CP (61, 62).

Importantly, mental health care is a clear need in the adult CP population, but access to adequate mental health care is not universal and is often contingent upon factors outside of the patient's control-including insurance coverage, availability within a geographical area, and fit between patient and provider. Increased capacity for mental health services, in part via implementation of telemedicine, is essential to even begin to address the need for support. Certain approaches to mental health care are likely to be differentially effective in adults with CP, although this has not yet been systematically studied. Use of a transdiagnostic treatment approach would appear to be an appropriate first line treatment, but future work is needed to assess efficacy and effectiveness.

## Conclusion

Adults with CP face barriers to health care at many levels, including the health system, the clinician, and the individual. They have many unique needs based on the changes in physiology that accompanied their growth and development, as well as social determinants of health common to them and many others with disabilities. Mental health issues are a prominent concern that will contribute to learned helplessness and interfere with self-management. More knowledge needs to be developed and disseminated in the form of evidence-based guidelines for health surveillance and appropriate interventions. Adults with CP should never be told “your CP is getting worse.” Rather, they are entitled to knowledge about the process of aging with CP and related risk factors, as well as coordinated care that leads to wellness, full participation, and excellent quality of life.

## Author Contributions

EH, DW, and MP conceived the concept of the review. EH, DW, BW-P, HH, MS, DR, and MP drafted the manuscript. CG provided critical feedback and contributed to the final manuscript. All authors contributed to the article and approved the submitted version.

## Funding

MP is the Charles E. Lytle, Jr. Research Professor for cerebral palsy research at the University of Michigan, and receives funding from the National Institute on Disability, Independent Living, and Rehabilitation Research (NIDILRR #90RTHF0001-01-00).

## Conflict of Interest

The authors declare that the research was conducted in the absence of any commercial or financial relationships that could be construed as a potential conflict of interest.

## Publisher's Note

All claims expressed in this article are solely those of the authors and do not necessarily represent those of their affiliated organizations, or those of the publisher, the editors and the reviewers. Any product that may be evaluated in this article, or claim that may be made by its manufacturer, is not guaranteed or endorsed by the publisher.
